# Improving the Efficacy of ERP-Based BCIs Using Different Modalities of Covert Visuospatial Attention and a Genetic Algorithm-Based Classifier

**DOI:** 10.1371/journal.pone.0053946

**Published:** 2013-01-14

**Authors:** Mauro Marchetti, Francesco Onorati, Matteo Matteucci, Luca Mainardi, Francesco Piccione, Stefano Silvoni, Konstantinos Priftis

**Affiliations:** 1 Department of General Psychology, University of Padova, Padova, Italy; 2 Department of Bioengineering, Politecnico di Milano, Milano, Italy; 3 Department of Electronics and Information, Politecnico di Milano, Milano, Italy; 4 Department of Neurophysiology, IRCCS San Camillo Hospital, Venezia-Lido, Italy; 5 Laboratory of Neuropsychology, IRCCS San Camillo Hospital, Venezia-Lido, Italy; University of Cambridge, United Kingdom

## Abstract

We investigated whether the covert orienting of visuospatial attention can be effectively used in a brain-computer interface guided by event-related potentials. Three visual interfaces were tested: one interface that activated voluntary orienting of visuospatial attention and two interfaces that elicited automatic orienting of visuospatial attention. We used two epoch classification procedures. The online epoch classification was performed via Independent Component Analysis, and then it was followed by fixed features extraction and support vector machines classification. The offline epoch classification was performed by means of a genetic algorithm that permitted us to retrieve the relevant features of the signal, and then to categorise the features with a logistic classifier. The offline classification, but not the online one, allowed us to differentiate between the performances of the interface that required voluntary orienting of visuospatial attention and those that required automatic orienting of visuospatial attention. The offline classification revealed an advantage of the participants in using the “voluntary” interface. This advantage was further supported, for the first time, by neurophysiological data. Moreover, epoch analysis was performed better with the “genetic algorithm classifier” than with the “independent component analysis classifier”. We suggest that the combined use of voluntary orienting of visuospatial attention and of a classifier that permits feature extraction *ad personam* (i.e., genetic algorithm classifier) can lead to a more efficient control of visual BCIs.

## Introduction

Farwell and Donchin [1] first investigated the possibility of participants to communicate by means of event-related potentials (ERPs; e.g., P300), without the involvement of the peripheral nervous system and the voluntary muscle activity. This is possible through brain-computer interfaces (BCIs), systems that permit users to translate their brain signals directly into commands for controlling external devices [2]. A BCI comprises a system for acquiring brain signals (e.g., an electroencephalograph for recording ERPs). Once acquired, brain signals are digitized and analyzed by specific algorithms for extracting specific features. Afterwards, these features are classified, and then they are translated into commands. Finally, these commands are executed by a device [3]. The execution of a command constitutes a feedback for the users about their performance. As a consequence, users must try to modulate their mental states (e.g., concentrate on the target stimulus and ignore the non-target ones) to obtain the desired effect on the device.

To date, the majority of the BCIs have relied on electroencephalographic (EEG) signals. The EEG technique has the advantage to be non-invasive, inexpensive, and suitable for the use at patients’ bedside. EEG-based BCIs can exploit users’ ability to modulate the sensorimotor rhythms (SMR) or the slow cortical potentials (SCP) [4]. Unfortunately, an efficient control of SMRs and SCPs requires long training, which can last for months [5]. The alternative solution is to exploit the EEG potentials that do not require long training for the users, because the signal is elicited by specific stimuli. This is the case of the BCI based on the P300 [6] or of the steady-state visually evoked potentials (SSVEPs) [7].

BCIs offer new perspectives regarding communication and control of devices for patients affected by severe motor impairment, such as patients with amyotrophic lateral sclerosis (ALS), who can be completely paralyzed. The ALS is a motor neurodegenerative pathology characterized by progressive paralysis resulting from selective death of both upper and lower motor neurones [8]. In the latest stages of the illness, ALS patients can show a clinical condition called the locked-in syndrome (LIS). LIS is characterised by quadriplegia, head muscles paralysis, and mutism. Nonetheless, consciousness is preserved [9]. Usually, the eye muscles are the last muscles that can be still controlled by LIS patients [10]. When the control of all the muscles is lost, the patients enter in the completely locked-in syndrome (CLIS), in which the communication abilities of the patients are completely absent. The use of brain signals might be the only way for giving a chance to LIS patients to communicate [11]. For this reason, in the last years the development of efficient BCIs for communication has been considered an important scientific and clinical challenge.

Although more than twenty years have passed since the first study [1], the P300 speller (i.e., the visual word spelling BCI), remains the most used and studied BCI. The P300 speller is composed by a 6×6 matrix of letters and numbers. Users have to concentrate their visuospatial attention on the target (i.e., a letter or an Arabic digit), while the brightness level of each row and column of the matrix is randomly and repeatedly changed. When the brightness of the row and column containing the target symbol is changed, the amplitude of the P300 is larger than when the brightness of the rows and columns containing non-targets is changed. Finally, the P300 with the expected feature (i.e., larger amplitude elicited by the target) is automatically detected by specific algorithms, and the target (i.e., letter or Arabic digit) is selected and displayed.

Several studies have been conducted on the P300 speller, to investigate the effects, on users’ performance, of different matrix sizes and inter-stimulus intervals (ITI [12], [13]), the effect of colour contrast between the stimuli and the background [14], and the effect of arranging the matrix depending on the psycholinguistic frequency of the English letters [15]. Most BCI studies, however, have been focused on the domains of signal processing and feature extraction. Different efficient techniques are available nowadays for signal classification: support vector machines (SVM [16]), stepwise linear discriminant analysis (SWLDA [17], [18]), Bayesian linear discriminant analysis [19], hidden Markov models [20], neural networks [21], and genetic algorithms (GA; [22]). In particular, the approach proposed by Dal Seno et al. [22] is appealing as it merges in a closed loop the feature extraction task (by using a GA) and the issue of the classification task (by using a logistic classifier).

In the classic approach [2], the feature extraction component is separated from the classification component: the extracted features are used to feed a classifier; in Dal Seno et al. [22] there was not an *a priori* feature set, but the “goodness” of the single feature was measured during the running of the GA itself, through the performances obtained by the logistic classifier. In this way, the two components of the system (feature extraction vs. classification) were in a closed loop, which was stopped when the obtained feature set did not further improve the classification performance. GAs have been already used in the BCI field, although in a different way from that in the study of Dal Seno et al. [22]. In the study of Boostani et al. [23] the best combination among different features and classifiers was sought for a motor-imagery task, whereas in the study of Citi et al. [24] a classifier, operating on P300 features, was selected by a GA.

Recently, it has been reported that good performances with the P300 speller are due to the participants’ possibility to move their eyes [25], [26]. Both Brunner et al. and Treder and Blankertz have reported that the classification accuracy of the P300 speller is not sufficient for communication, if participants cannot perform eye movements (even healthy participants). Thus, the fact that the P300 speller performance depends on eye movements can be a critical obstacle for the use of visual BCIs by CLIS patients [27]. Nonetheless, the use of visual BCIs seems to be still possible by LIS patients who might have some residual eye movements [27], [28]. To overcome this problem, two different solutions have been proposed. The first solution was to develop P300-BCI systems based on other senses, such as auditory BCIs [28]–[32] and tactile BCIs [33]. The second solution was to design visual BCIs based on the covert (i.e., without eye movements) orienting of visuospatial attention [19], [34], [35].

In our recent study [35], we tested a P300-based BCI for controlling the movement of a cursor on a screen with a four choice interface [36], in a covert visuospatial attention condition. The aim of Marchetti et al. [35] was to investigate whether there was an advantage in implementing the principles of covert orienting of visuospatial attention, described by Posner [37], on these interfaces. Many studies in the last four decades (for a comprehensive, recent review, see [38]) have suggested that visuospatial attention can be oriented by two types of cues: peripheral cues, which elicit an automatic orienting of visuospatial attention, and central cues, which activate voluntary orienting of visuospatial attention.

We investigated the possibility to modulate the performance of an ERP-based BCI system, by designing and implementing three new interfaces (see [35]), in which participants were required to perform covert orienting of visuospatial attention [37], [39]. The first interface (“Arrows”) was similar to that used by Piccione et al. [36]. The second interface (“Auto”) was designed by implementing the automatic orienting of visuospatial attention. The third interface (“Vol”) was designed by implementing the voluntary orienting of visuospatial attention. Note that also the interface proposed by Piccione et al. was implicitly based on the automatic orienting of visuospatial attention. By using on-line classification, Marchetti et al. showed that good performance could be reached using visual interfaces controlled without eye movements. Furthermore, it was reported that the interface based on the voluntary orienting of visuospatial attention could yield better performance than those based on the automatic orienting of visuospatial attention.

To investigate whether the findings of Marchetti et al. [35] depended on the classification system they had used, in the present study we performed an offline reclassification of the original EEG data. In the previous study the online analysis of the epochs was performed by means of Independent Component Analysis (ICA) and of subsequent fixed features extraction and support vector machines (SVM) classification [40]. In the present study we performed offline analysis by means of a GA that permits to retrieve the relevant features of the signal, which can be classified by means of a logistic classifier [41]. Thus, we tested whether the effects reported by Marchetti et al. [35] depended on the specific classification system used, and whether the offline classification performed with the GA could improve classification with respect to the previously used classification system (i.e., “ICA classifier”) [40], [35]. In addition, we used a measure of performance that takes into account the specific characteristics of our visual interfaces. Indeed, it has been reported that the same performance level, achieved by means of different BCIs (e.g., the four choices interface of Piccione et al. [36]; or the 3×3/6×6 choices interfaces tested by Sellers et al. [13]), can be associated with different BCI-control efficacy [42], [43]. We used the F-measure (see below) instead of the classic accuracy measure (i.e., the percentage of correct classifications [2]), because of the unbalanced number of targets and non-targets in our study. We could have artificially balanced the training and the testing sets; but, besides being not realistic, this approach does not take into account the presence of false positives and false negatives [44]. Finally, we tested for the first time the effects of the three interfaces on the participants’ brain potentials, as a function of the two classification approaches (i.e., “ICA classifier” vs. “GA classifier”). In summary, the novelty of the present study, with respect to that of Marchetti at al. [35], consisted in the use of the GA and in the implementation of the F-measure. In addition we analysed not only performance but also the ERPs, for interpreting the classification results under the light of the possible different morphology of the ERPs elicited by the three interfaces.

## Methods

### 2.1 Participants

Twelve healthy participants with normal or corrected-to-normal vision took part in the study (mean age: 37 years; range: 20–61 years; 5 males). The study was performed in accordance with the Declaration of Helsinki principles. All participants gave their oral informed consent to participate in the study. The research project (including the use of oral informed consent) was approved by the Ethical Committee of the IRCCS San Camillo Hospital, Venice-Lido. Oral consent was used given that all participants’ data remained anonymous. Upon their own request, participants could interrupt their participation to the experiment at any moment, without any negative consequences for them, and without providing explanations regarding their withdrawal from the study. Identity information of all participants who gave oral informed consent was documented in a separate file. This process was witnessed by both the participants and the experimenters.

### 2.2 Apparatus, Stimuli, and Procedure

**Figure 1:**
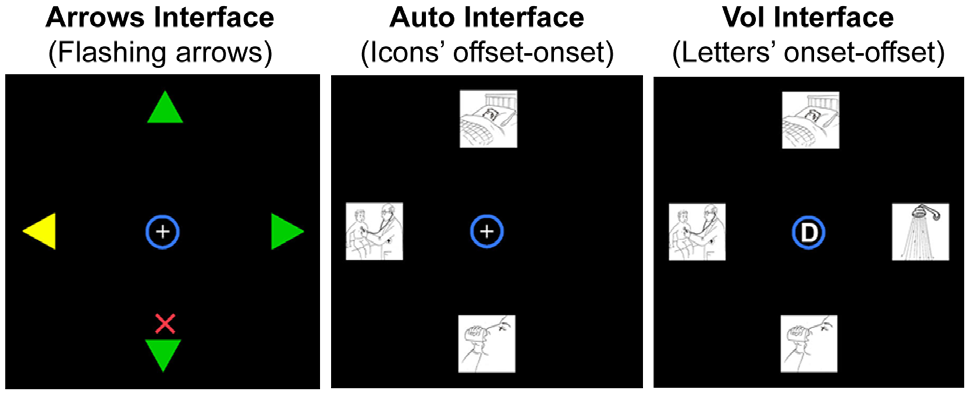
The three interfaces.

The experiment took place in a sound-attenuated chamber. Participants sat in an adjustable chair in front of a computer screen (HP L1906T Flat Panel LCD Screen; dimension: 38×30.5 cm; refresh frequency: 60 Hz; resolution: 1024×768), with their head positioned on a chinrest fixed on the table. The distance between the center of screen and the participant’s eyes was 57 cm. At a distance of 57 cm between the center of screen and the participant’s eyes, 1° of visual angle corresponds to the size of 1 cm on the monitor. Three interfaces (Figure 1) were presented to all participants: two designed on the basis of the principle of the automatic orienting of visuospatial attention and one designed on the basis of the principle of the voluntary orienting of visuospatial attention. All interfaces were based on the Piccione et al.’s [36] paradigm, where participants had to control the movement of a cursor to reach a target position by paying attention to peripheral cues. The three interfaces have been extensively described in Marchetti et al. [35].

In each interface all stimuli were displayed against a black background. Each interface comprised a fixation point (i.e., a white cross presented in the center of the screen) and a cursor placed in the center of the screen (i.e., a blue circle measuring 1° in diameter). During the experimental sessions with all the interfaces, participants were required to maintain their gaze on the fixation point and to avoid head and eye movements, while their EEG was recorded.

**Figure 2:**
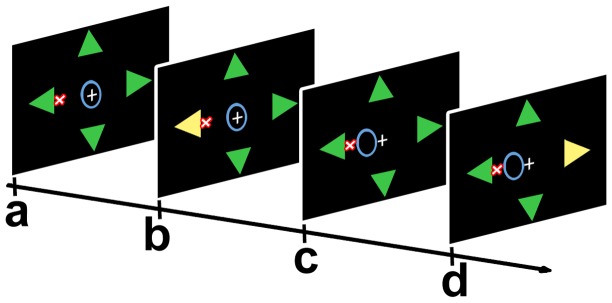
The “Arrows” interface. Scheme of a “target” trial: a) initial situation of a session: the fixation point (white cross), the cursor (the circle initially placed at the center, colored in blue), and the four arrows were displayed on the monitor; b) one arrow changed color for 150 ms; c) if the classifier recognized the “target” ERP pattern, then the cursor was moved one step towards the spatial position of the arrow that had changed color; d) next trial (ITI 2.5 sec): an arrow in a different spatial position changed color. Note that the target was the X placed close to one of the arrows, which was colored in red.

The “Arrows” interface (Figure 2) was similar to that designed by Piccione et al. [36], and used a stimulation paradigm that elicited automatic orienting of visuospatial attention. Four arrows were presented in the periphery of the screen at a distance of 7° from the fixation point. Each arrow indicated one out of four possible directions: above, right, below, and left. On each trial, a red “X” indicating the target position was displayed close to a specific arrow.

For eliciting brain potentials, we used a fast change of the color of each arrow from green to yellow and then back to green (color change duration: 150 ms; overall event probability for each arrow: 25%). A trial was defined as the time elapsed between the consecutive color changes of two arrows. Participants were required to pay attention to the arrow close to the red X (target) and to ignore the other three arrows (non targets), to control the movement of the cursor for reaching the red X.

**Figure 3:**
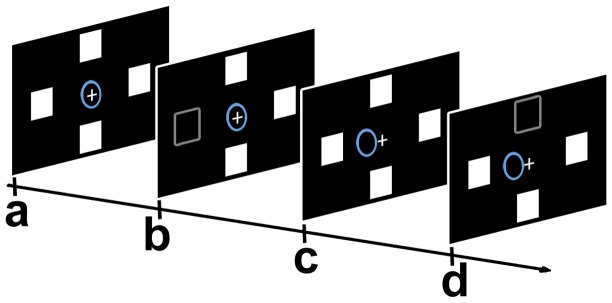
The “Auto” interface. Scheme of a “target” trial: a) initial situation of a session: the fixation point (white cross), the cursor (the circle initially placed at the center, colored in blue), and the four icons were displayed on the monitor; b) one icon disappeared (represented by the grey shape) for 75 ms and reappeared in the same position; c) if the classifier recognized the “target” ERP pattern, then the cursor was moved one step towards the spatial position of the icon which disappeared; d) next trial (ITI 2.5 sec): an icon in a different spatial position disappeared.

The “Auto” interface (Figure 3) used a stimulation paradigm that elicited automatic orienting of visuospatial attention. Instead of the arrows, four icons were presented in the periphery of the screen at a distance of 7° from the fixation point. The icons were four squared (side: 3.5°), black-and-white drawings depicting everyday life activities (eating, drinking, etc.). The drawings had been adapted from a battery for the assessment of aphasic disorders [45].

For eliciting brain potentials, we used a brief offset of one icon (duration: 75 ms; overall event probability for each icon: 25%) and its onset in the same position. A trial was defined as the time elapsed from the offset of an icon to the offset of the next icon. Participants were required to pay attention to the target icon, previously indicated by the examiner, and to ignore the remaining three non-target icons, in order to control the movement of the cursor for reaching the target.

**Figure 4:**
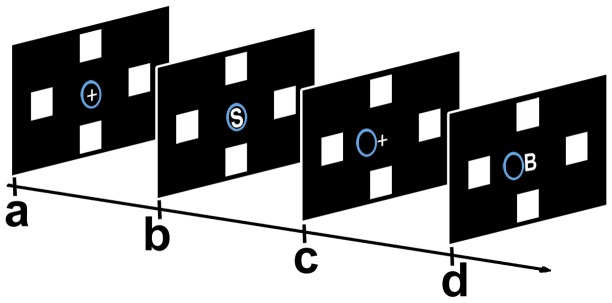
The “Vol” interface. Scheme of a “target” trial: a) initial situation of a session: the fixation point (white cross), the cursor (blue circle), and the four icons were displayed on the monitor; b) one capital letter indicating a spatial position appeared for 900 ms; c) if the classifier recognized the “target” ERP pattern, then the cursor was moved one step towards the spatial position indicated by the letter; d) next trial (ITI 2.5 sec): a capital letter indicating a different spatial position appeared.

In the “Vol” interface (Figure 4) we used a stimulation paradigm that activated a voluntary orienting of visuospatial attention. In this interface we used the same display as that used for the “Auto” interface.

For eliciting brain potentials, on each trial we presented at the fixation point one out of four capital letters (duration: 900 ms, overall event probability for each letter: 25%), while the four icons remained always on the screen. A trial was defined as the time elapsed from the onset of a letter to the onset of the next letter. Each letter was the initial letter of an Italian spatial directional word (i.e., “A”: *alto* = above, “B”: *basso* = below, “S”: *sinistra* = left, “D”: *destra* = right), each indicating the position of a specific icon. Participants were required to attend to the onset of the letter defining the spatial position of the target icon, which was indicated by the examiner before each session, and to ignore the other three letters.

The order of the events for eliciting the brain potential was semi-random in all the interfaces. That is, within each block of four consecutive trials, each of the four possible events (i.e., “Arrows” interface = brief color change of one arrow, “Auto” interface = offset-onset of one icon, “Vol” interface = onset-offset of one letter) occurred randomly. The first trial of the next block could have been either the same one or a different one from that of the last trial of the preceding block. The ITI was 2.5 s. The initial distance between the starting-point of the cursor (i.e., center of the screen) and each of the targets, encompassed four discrete steps in all interfaces. Each time the classifier detected the target ERP in the EEG epoch, following one of the four possible events, the cursor moved one step on the screen accordingly to the direction of the event that elicited the ERP. On the contrary, each time the classifier detected a non-target ERP in the EEG epoch, following one of the four possible events, the cursor was not moved. Thus, at least four correct classified epochs following the target event were required to reach the target icon (i.e., true positive case). Note that even if the classifier detected a target ERP following a non-target event, the cursor was moved one step towards the direction of the non-target event (i.e., false positive case). A session was defined as the sequence of trials needed to reach the target, to reach one of the non-targets, or after 92 trials elapsed without reaching a target or a non-target. As a consequence of the interface design and the single epoch classification, the number of trials presented during the BCI sessions was different among the participants and within the sessions performed by each participant (range 13–92 trials).

**Figure 5:**
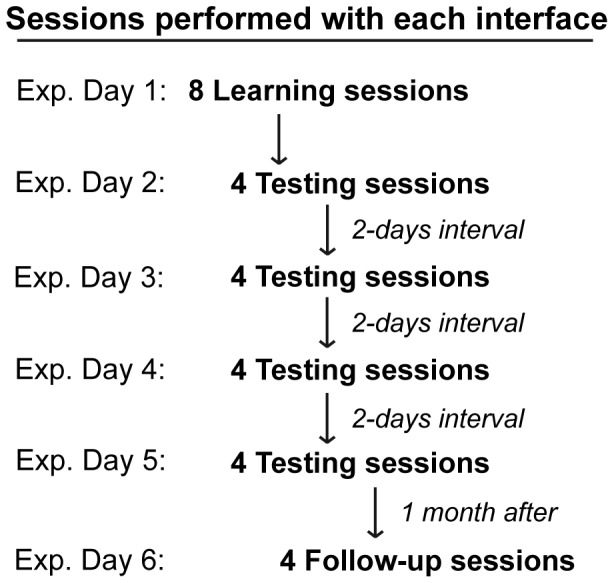
Experimental procedure.

For each interface participants performed 8 learning sessions in the first experimental day, 16 testing sessions that were distributed over the following ten days, and four follow-up sessions which took place, on average, 27 days after the last testing sessions (Figure 5).

The learning sessions were characterized by a “perfect feedback”, provided to participants by a correct movement of the cursor, which did not depend on the online classification system. This was necessary for collecting the first sample of epochs related to target and non-target trials, in order to prepare the online classifier for the first day of the testing sessions. In contrast, during the testing sessions the cursor was moved only as a response to participants’ brain waves, once classified as target ERPs. Thus, in each testing and follow-up session the number of trials was different for each participant, depending upon the performance of the classifier and the ability of the participant to control the cursor movements. Within an experimental day, the position of the targets was different for each session. The target positions in the follow-up sessions were the same as those in the sessions of the last testing day. The order of target positions was counterbalanced across testing sessions. The order of presentation of the three interfaces was counterbalanced across participants.

### 2.3 Electrophysiological Data Acquisition and Processing

On each trial the EEG was recorded. Recording electrodes were placed according to the International 10–20 System at Fz, Cz, Pz, and Oz. The Electrooculogram (EOG) was recorded from a pair of electrodes below and laterally to the left eye. All electrodes were referenced to the left earlobe and the ground was on Fpz. Impendence was lower than 5 kΩ. The five channels were amplified, band-pass filtered between 0.15 Hz and 30 Hz, and digitized (with a 16-bit resolution) at 200 Hz sampling rate. Each ERP epoch, synchronized with the stimulus, began 500 ms before the stimulus onset and ended 1000 ms after the cue (total duration: 1500 ms). Thus, after each cue presentation the system recorded a matrix of 300 samples per 5 channels, available for online and offline data processing.

#### 2.3.1 “ICA classifier” data analysis

To control online the BCI system, we used a classification algorithm that has been extensively described elsewhere [35], [36], [40]. Before each testing day and for each of the three interfaces a different classifier was trained and adapted ad personam through a three-step procedure: Independent Component Analysis (ICA) decomposition, fixed features extraction, and support vector machine (SVM) classification. The ICA decomposition was used for splitting up the EEG signals into statistically independent sources of signal [46], [47], with the specific hypothesis that one of the sources reflected the ERP. Then the source that was more similar to the target ERP was automatically selected using a fuzzy method [48]. On the basis of the selected source, a single-sweep normalized data set was obtained for each trial of the testing sessions, and it was used for feature extraction [49]. The extracted features encompassed a set of 78 values representing a concise description of the ERPs. The extracted features were used for the classification of the testing session trials with a SVM classifier.

The SVM classifier was updated after each testing day with a 20-fold cross-validation procedure, except for the epochs of the last session [50]. The 80% of the remaining epochs were randomly selected as training set, whereas the 20% composed the testing set. ERP epochs with artifacts greater than 100 µV, with regard to each channel’s activity (including EOG), were excluded from each training set [51]. All available ERP epochs were analyzed for each testing set. The epochs of the last session were used to perform a further validation of the updated SVM classifier. After the last testing session, no other classifier updating was performed. Thus, the classifier used in the follow-up sessions was the same as that of the last testing sessions. The three-step classification procedure was applied during online operations to each single sweep synchronized with the cue. The output of the SVM classifier was converted into a binary value (1 = target ERP; 0 = non target ERP) to control each movement of the cursor.

#### 2.3.2 “GA classifier” data analysis

For the offline classification of target vs. non-target ERPs, we used a method that combines a GA for both feature extraction and selection, and a logistic classifier for classification. A detailed description of the method can be found in Dal Seno et al. [22]; here only the main aspects are reported.

GAs belong to the class of evolutionary algorithms (i.e., optimization algorithms inspired by the theory of evolution) [52]. In particular, in a GA the solutions of the optimization problem are coded in strings called chromosomes: the best chromosomes are selected, combined together, and modified in a process which imitates that of evolution, including mutation, cross-over, and natural selection. Generation-by-generation, the best solution will emerge from a population of sub-optimal solutions [53]. In our implementation, each chromosome encoded the set of features, to be used by the logistic classifier for the classification of target vs. non-target ERP epochs. Each feature was computed by the dot product between the EEG signal and a weighting function coded in one gene. Different weighting schemas were potentially similar to the possible shapes of kernel, in a kernel smoothing approach. In the present study we used the Gaussian weighting function kernel. The gene was characterized by four parameters: two parameters characterized the Gaussian curve (the timing of the Gaussian peak within the epoch, and its width), one parameter identified the EEG lead used, and the last parameter activated/deactivated the gene (i.e., the parameter stated whether the related feature should be used for classification or not). The length of the single chromosome (i.e., the number of its genes) was not defined a priori. Indeed it might change from generation to generation [54]. By combining several Gaussian functions we obtained a Radial Basis approximation of the original signal, as was also done in Karjalainen et al. [55].

A constant population of 120 individuals, randomly initialized, was used. The maximum number of generations was set to 20.Tthe evolution was stopped if both the maximum value and the average value of a performance metric (i.e., the F-measure, defined below) did not increase for at least 4 generations. Tournament selection with elitism was used as the selection criterion [53]. A tournament size of 10 chromosomes and an elitism of 2 individuals were used for each generation. After selection, individuals underwent crossover and mutation. Crossover was applied to pairs of chromosomes randomly chosen with a probability of 0.7.The chromosomes were randomly divided in two segments (without breaking any gene), and then the four parts were recombined. Mutation was applied to any single element of the gene with a probability of 0.005.Mutation consisted in small perturbations of its value. The output of our GA consisted in a set of chromosomes. It included all the chromosomes with a performance above 99% of the maximum performance value obtained during the whole evolution. In our implementation, the features extracted from the GA were used as input for a logistic classifier [56]. For each single interface, learning and testing sessions (Section 2.2) were used as training set for the logistic classifier, whereas last testing and follow-up sessions (Section 2.2) were used as testing set. To avoid over fitting in the optimization of features by the GA, k-fold cross-validation (k = 4) was used [57]. GA parameters were not optimized for our specific dataset. The parameters’ values used in the present study were the same as those used in previous studies [22], [41]. Although it could be possible to improve the GA’s speed and its final solution, by optimizing the choice of the abovementioned parameters, this was not necessary for the scope of the present study.

#### 2.3.2 Artifactual epochs detection

The percentage of ERP epochs with amplitude values greater than 75 µV was calculated. This analysis was performed to obtain a measure of the eye-movement artifacts for the Testing sessions and the Follow-up sessions of each interface. The criterion of ±75 µV was chosen in order to detect the artifacts due to eye-blinks [58] and in order to detect gaze shifts greater than 5° of visual angle. In fact, a bipolar EOG recording permits to detect deflections of about 16 µV for each degree of eye movement [59]. The distance between the fixation point and the center of the icons (i.e., “Auto” and “Vol” interfaces) or the barycenter of the arrows (i.e., “Arrows” interface) was of 7° of visual angle. The cut-off level of ±75 µV permitted us to detect whether there were artifacts in the epochs, because of gaze shifts from the center towards one of the icons or arrows displayed in the periphery of the interfaces (or viceversa).

### 2.4 Experimental Design

Independent variables were manipulated within an experimental design for repeated measures. We manipulated the following independent variables to test whether there was a non-homogeneous distribution of eye artifacts because of gaze-shifts or blinks among the experimental conditions: Interface with three levels (“Arrows”, “Auto”, “Vol”) and Session with two levels (Testing sessions, Follow-up sessions). The dependent variable measured was the percentage of ERP epochs with amplitude values greater than 75 µV.

The independent variables manipulated for testing the effects on BCIs’ performance were: Classifier with two levels (“ICA classifier”, “GA classifier”), Interface with three levels (“Arrows”, “Auto”, “Vol”), and Session with two levels (Testing sessions, Follow-up sessions). To assess classification performances, the F-measure [44] was chosen as the dependent variable. Most commonly used in Information Retrieval, F-measure is the harmonic mean of recall (Re) and precision (Pr). Re is the rate of the number of correct target classification with respect to the number of true target epochs (true positive epochs/total target epochs), whereas the Pr is the rate of correct target classification with respect to the epochs that have been labeled as target by the classifier (true positive epochs/[true positive epochs+false positive epochs]). The traditional F-measure (or balanced F-measure) equally weights precision and recall and it is defined as (1):
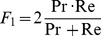
(1)


We have used F-measure, instead of the classic accuracy measure [2], mainly because of the unbalanced number of targets and non-targets in our experiments. We could have artificially balanced the training and the testing sets. Besides being not realistic, this approach does not take into account the presence of false positives and false negatives.

The independent variables manipulated for testing the experimental effects on the ERP were: Interface with three levels (“Arrows”, “Auto”, “Vol”), Session with two levels (Testing sessions, Follow-up sessions), Channel with four levels (Fz, Cz, Pz, and Oz), and Trial Class with two levels (Target, Non-target). The dependent variables were the amplitude of the P300 component and the amplitude of the late negative component (LNC). The amplitude of the P300 was defined as the averaged ERP amplitude from 300 to 500 ms with respect to a baseline (i.e., the average amplitude of the epoch from 500 ms before the stimulus onset to the zero, the point of the stimulus onset). The amplitude of the LNC was defined as the averaged ERP amplitude from 500 to 900 ms with respect to the baseline. The time windows used for the amplitude definition were identified through visual inspection of the grand average ERP by the experimenters.

## Results

Data were subjected to Analyses of Variance (ANOVA) for repeated measures. The Greenhouse-Geisser correction coefficient (ε) is reported when the assumption of sphericity has been violated.

### 3.1 Eye-movement Artifacts Analysis

The percentage of eye-movement artifacts was lower than 15% in all the conditions. The percentage of eye-movement artifacts for each participant was subjected to ANOVA for repeated measures. Both the main effect of the Interface and the main effect of the Session were not significant, *F*(2,22) = .65, *p* = .531, and *F*(1,11) = .64, *p*>.05, respectively. Also the interaction effect between Interface and Session was not significant, *F*(2,22) = .46, *p*>.05.

### 3.2 Classification Performance Analysis

**Figure 6:**
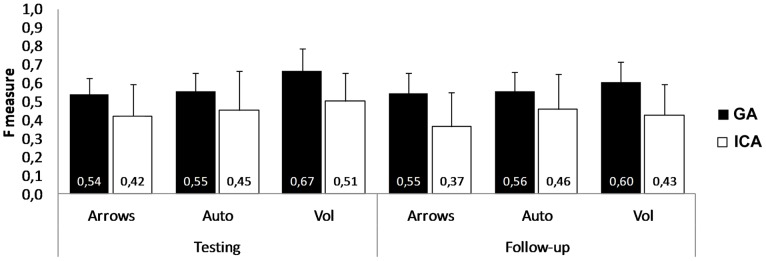
Classification results. Means and Standard Deviations of the performance (F-measure) of online (ICA) and offline (GA) classification.

The results of the classification performance with the “ICA classifier” and with “the GA classifier” for the Testing and Follow-up sessions of each interface are reported in Figure 6.

There was a main effect of the Classifier *F*(1,11) = 24.22, *p*<.001, because of a better classification performance of the “GA classifier” (M = 57.85, SD = 11.24), than that with the “ICA classifier” (M = 43.93, SD = 17.87). Participants’ performance was significantly modulated as a function of the Interface, *F*(2,22) = 13.99, *p*<.001. Post-hoc comparisons, corrected with Bonferroni, revealed that participants reached higher performance with the “Vol” interface (M = 55.16, SD = 16.38), than with both the “Auto” (M = 50.61, SD = 16.31; *p*<.05) and with the “Arrows” (M = 55.16, SD = 16.38; *p*<.001) interfaces. The comparison between the “Auto” and the “Arrows” interfaces was not significant (*p*>.05). Moreover, participants’ performance was lower in the Follow-up sessions (M = 49.43, SD 16.42), than in the Testing sessions (M = 52.35, SD = 16.43), Session, *F*(1,11) = 5.97, *p*<.05.

The Classifier by Interface interaction was significant, *F*(2,22) = 5.63, *p*>.05. To further investigate this interaction effect, an analysis of the simple effect of the Interface within each level of the Classifier factor was performed. The simple effect of the Interface was significant when the epoch classification was performed by means of the “GA classifier”, *F*(2,22) = 14.22, *p*<.001. Post-hoc comparisons, corrected with Bonferroni, showed that higher performance was associated with the “Vol” interface (M = 63.6, SD = 12.01) than with both the “Auto” (M = 55.63, SD = 10.13; *p*>.05) and the “Arrows” (M = 54.31, SD = 9.53; *p*<.001) interfaces. On the contrary, there was no significant difference between the “Auto” and “Arrows” interfaces (*p*>.05). The simple effect of the Interface was significant also when the epoch classification was performed by means of the “ICA classifier”, *F*(2,22) = 8.57, *p*<.01. This Interface effect was due to a significantly lower classification performance associated with the “Arrows” interface (M = 39.5, SD = 17.62), than with both the “Auto” (M = 45.58, SD = 19.71; *p*<.05) and the “Vol” (M = 46.72, SD = 15.98; *p*<.01) interfaces. There was no significant difference between the “Auto” and the “Vol” interfaces (*p*>.05).

The Interface by Session interaction was also significant, *F*(2,22) = 4.98, *p*<.05. To further investigate this interaction effect, an analysis of the simple effect of the Session for each interface was performed. There was a significant reduction in the classification performances in the Follow-up session (M = 51.6, SD = 12.36), with respect to the Testing sessions (M = 58.71, SD = 13.32; *p*<.01) of the “Vol” interface. In contrast, the comparisons between the Follow-up and Testing sessions within both the “Auto” and “Arrows” interfaces were not significant (*p*>.05).

Both Classifier by Session interaction and the Classifier by Interface by Session interaction were not significant, *F*(1,11) = 1.16, *p*>.05 and *F*(2,22) = 1.14, *p*>.05, respectively.

### 3.4 P300 Amplitude Analysis

The ANOVA results for the mean amplitude of the P300 are shown in Table 1. For reason of clarity, only the results relevant for the hypotheses of the present study were extensively reported in the paragraph below, especially those where the Trial Class factor was involved.

**Table 1:** Results of the ANOVA for the P300 amplitude.

Factors	*F*	Df	*p*	ε
Interface	.34	(2, 22)	.62	.64
Channel	4.95	(3, 33)	.006	–
Session	3.07	(1, 11)	.11	–
Trial Class	9.76	(1, 11)	.01	–
Interface × Channel	1.24	(6, 66)	.31	.29
Interface × Session	.89	(2, 22)	.42	–
Channel × Session	.45	(3, 33)	.71	.43
Interface × Trial Class	2.84	(2, 22)	.08	–
Channel × Trial Class	2.89	(3, 33)	.05	–
Session × Trial Class	.59	(1, 11)	.68	–
Interface × Channel × Session	.77	(6, 66)	.5	.43
Interface × Channel × Trial Class	.59	(6, 66)	.74	.33
Interface × Session × Trial Class	1.94	(2, 22)	.17	–
Channel × Session × Trial Class	.99	(2, 22)	.41	–
Interface × Channel × Session × Trial Class	2.86	(6, 66)	.8	.56

The main factors and the interactions are reported in the first column. The *F* values, the related degrees of freedom (df), the associated *p* values (in bold are reported those which are <.05), and the Greenhouse-Geisser correction coefficient (ε) are reported in the following columns.

**Figure 7:**
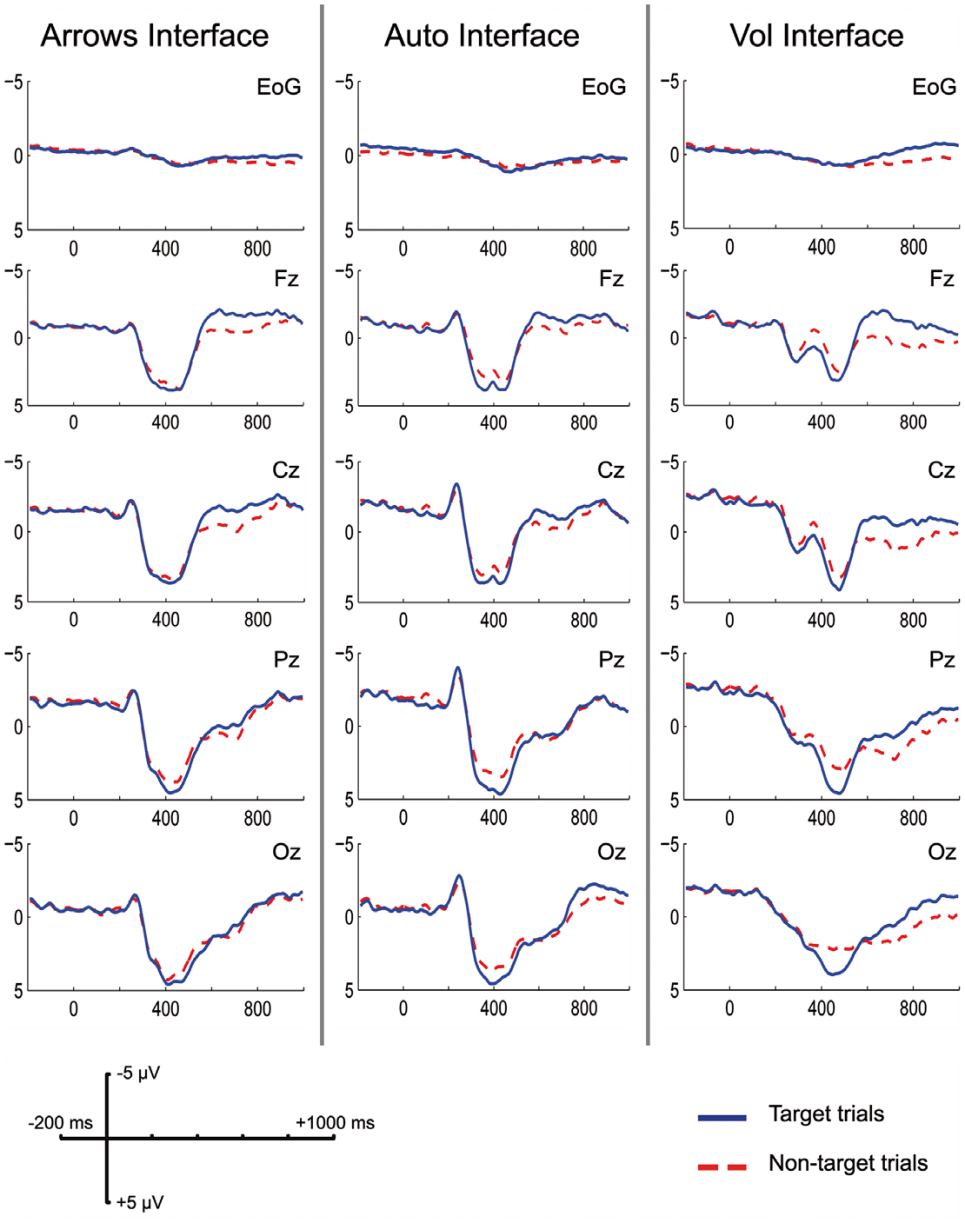
ERPs grand average. Grand average of the ERPs elicited by the three interfaces on the last testing and follow-up sessions.

The main effect of the Trial Class was significant, *F*(1,11) = 9.76, *p*<.05. A larger P300 was elicited following the Target trials (M = 4.83 µV, SD = .86) than following the Non-target trials (M = 3.32 µV, SD = .45). The distribution of the P300 increased in amplitude from the frontal to the posteriors sites (Channel, *F*(3,33) = 4.95, *p*<.01; see Figure 7). The three interfaces elicited P300s of similar amplitudes on Target and Non-target trials. In fact, the interaction Interface by Trial Class (*F*(2,22) = 2.84, *p*>.05) and Interface by Channel by Trial Class (*F*(6,66) = .59, *p*>.05, ε = .33) were not significant. Moreover, none of the effects involving the Session factor was significant.

To investigate whether the differences between the interfaces’ performance found in the EEG data classification were due to differences in P300 amplitude, a Target vs. Non-target trials planned contrast was performed within each interface. There was a significant difference in P300 amplitude in the “Vol” interface (Target: M = 4.99 µV, SD = 2.36; Non-target: M = 2.78 µV, SD = 1.32; *t*(11) = 4.98, *p*<.001), but not in the “Auto” (Target: M = 4.78 µV, SD = 3.7; Non-target: M = 3.54 µV, SD = 1.94; *t*(11) = 1.91, *p*>.05) and in the “Arrows” (Target: M = 4.72 µV, SD = 3.33; Non-target: M = 3.61 µV, SD = 1.78; *t*(11) = 1.88, *p*>.05) interfaces (see Figure 7).

### 3.4 LNC Amplitude Analysis

The ANOVA results for the mean amplitude of the LNC are shown in Table 2. Only the results relevant for the experimental questions of the present study were extensively reported, with particular reference to the Trial Class factor.

**Table 2:** Results of the ANOVA for the LNC amplitude.

Factors	*F*	Df	*p*	ε
Interface	3.97	(2, 22)	.034	–
Channel	4.63	(3, 33)	.008	.55
Session	.02	(1, 11)	.89	–
Trial Class	21.43	(1, 11)	.001	–
Interface × Channel	1.07	(6, 66)	.39	.26
Interface × Session	.14	(2, 22)	.87	–
Channel × Session	.82	(3, 33)	.49	.44
Interface × Trial Class	8.97	(2, 22)	.001	–
Channel × Trial Class	11.05	(3, 33)	.002	.49
Session × Trial Class	.8	(1, 11)	.39	–
Interface × Channel × Session	.89	(6, 66)	.5	.28
Interface × Channel × Trial Class	1.15	(6, 66)	.34	.45
Interface × Session × Trial Class	.69	(2, 22)	.93	–
Channel × Session × Trial Class	.28	(2, 22)	.84	–
Interface × Channel × Session × Trial Class	.59	(6, 66)	.59	.42

The main factors and the interactions are reported in the first column. The *F* values, the related degrees of freedom (df), the associated *p* values (in bold are reported those which are <.05), and the Greenhouse-Geisser correction coefficient (ε) are reported in the following columns.

There was a larger negativity in the last portion of the epochs on the Target (M = −2.22 µV, SD = .51) than on the Non-target trials (M = −.05 µV, SD = .2), Trial Class, *F*(1,11) = 21.43, *p*<.01. This difference was significantly larger in the frontal site and progressively decreased towards the posterior sites along the midline (see Figure 7), Channel by Trial Class *F*(3,33) = 11.05, *p*<.01, ε = .49. The Interface by Trial Class interaction was significant, *F*(2,22) = 8.97, *p*<.01, revealing that the LNC amplitude related to the Target and Non-target trials was differently modulated among the three interfaces. To further investigate this interaction effect, two separate ANOVAs were performed for testing the simple effect of the Interface on each level of the Trial Class (i.e., Target and Non-target). No different modulation in LNC amplitude was found on the Target trials, *F*(2,22) = .24, *p*>.05. In contrast, there was a significant effect of the Interface on the Non-target trials, *F*(2,22) = 25.56, *p*<.001. Post hoc comparisons, corrected with Bonferroni, showed that the amplitude values related to Non-target trials on the “Vol” interface (M = .87 µV, SD = .29) were different from those on the “Auto” (M = −.47 µV, SD = .18, *p*<.001) and from those on the “Arrows” (M = −.57 µV, SD = .22, *p*<.01) interfaces. In contrast, there was no significant difference between the “Auto” interface and the “Arrows” interface, *p* = 1.

As for the P300, planned contrasts on Target vs. Non-target trials were performed within each interface. There was a significant difference between the two levels of the Trial Class in each interface: the “Vol” interface (Target: M = −2.24 µV, SD = 2.29; Non-target: M = .87 µV, SD = 1.01; *t*(11) = −5.87, *p*<.001), the “Auto” interface (Target: M = −2.42 µV, SD = 1.64; Non-target: M = −.47 µV, SD = .64; *t*(11) = −4.07, *p*<.005) and the “Arrows” interface (Target: M = −1.98 µV, SD = 1.99; Non-target: M = −.57 µV, SD = .78; *t*(11) = −2.53, *p*<.05).

## Discussion

We analyzed whether the performances obtained by 12 healthy participants using three new interfaces in an ERP-based visual BCI [35] were influenced by the specific classification system. For this purpose, we performed an offline classification with a GA for the features extraction and a logistic classifier for epoch categorization [22]. The F-measure was calculated and used as the dependent variable for both online and offline classifications. We used the F-measure because it is a performance index that takes into account the unbalanced number of targets and non-targets. By using the F-measure, we overcame some intrinsic limitations of the classic measures used for testing discrete BCIs [41], [42].

The results of the “GA classifier” analysis revealed different performances among the three interfaces. Participants reached better accuracy with the “Vol” interface than with both the “Auto” and the “Arrows” interfaces. These results are in line with those reported by Marchetti et al. [35], who used: a) online classification procedure (i.e., “ICA classifier”) with a fixed features extraction algorithm, and b) the classic accuracy as the performance measure (i.e., the percentage of correct classified trials on the total number of trials [2], [36]). Our data support the hypothesis, that independently of the classification system and of the specific measure (online/classic accuracy measure vs. offline/F-measure), the “Vol” interface (guided by voluntary orienting of visuospatial attention) leads the users to reach a better control of the cursor movement. In accordance with the results of the offline “GA classifier” analysis using the F-measure, we found a different modulation of the LNC amplitude on non-target trials among the three interfaces. That is, the LNC amplitude was lower in the “Vol” interface than in the “Auto” and in the “Arrows” interfaces, revealing that voluntary orienting of visuospatial attention can be more efficient in inhibiting brain activity related to non-target events.

On the contrary, the results obtained with the “ICA classifier” analysis by means of the F-measure showed that participants’ performance was lower in the “Arrows” interface than in the other two interfaces. No difference was found between the “Auto” and “Vol” interfaces. Nevertheless, the different results obtained with the “ICA classifier” analysis might depend on the use of fixed features extraction, which seemed to be less sensible in detecting the differences among the interfaces.

Being the two classifiers tested on exactly the same data, a fair comparison is possible. Performance with the “GA classifier”, outperformed that with the “ICA classifier”. The higher performance obtained and the smaller standard deviation (see Figure 6), suggest that the use of the “GA classifier” might be a better solution for online epoch categorization within our BCI system.

We did not control the position of the participants gaze during the sessions with an eye-tracker system, and this might represent a limit of the present study. It could be argued that the Interface effect we found could be due to the participants’ possibility to move their gaze towards different spatial positions on the monitor during the BCI sessions. Nonetheless, the analysis that we performed on the percentage of trials with eye-movements artifacts, supported the idea that the participants did not use different “eyes-movement strategies” for controlling the three interfaces. The percentage of epochs with eye-movement artifacts in all the interfaces was lower than the 15% of the total number of trials. Furthermore, none of the participants reported any problem in maintain the gaze at the fixation point during the BCI sessions.

In summary, the results showed that the control of a visual ERP BCI is possible in a condition of covert orienting of visuospatial attention that is particularly relevant for patients, whose eye muscle control is impaired. Moreover, subtle differences in interface design, such as the implementation of the voluntary and automatic orienting of visuospatial attention principles, produced significant differences on the ERP elicited and, consequently, on BCI performance. This result represents a further evidence of the fact that the implementation of cognitive principles on BCI design and development can modulate the underlying brain signals, leading to advantages in device control for the user. Nonetheless, to take full advantages of such design implementations, classifying systems which do not operate on *a priori* feature extraction are suggested. For this purpose, the use of genetic algorithms might represent an efficient *ad hoc* solution for detecting the most relevant features deriving from both distinct interface modulation and interpersonal brain signal differences.
